# No difference in bacterial contamination of hip capsule sutures and control sutures in hip arthroplasty surgery

**DOI:** 10.1186/s13756-023-01305-0

**Published:** 2023-09-14

**Authors:** Thomas J. A. van Schaik, Maurits P. A. van Meer, Lex D. de Jong, Jon H. M. Goosen, Matthijs P. Somford, Job L. C. van Susante

**Affiliations:** 1 Department of Orthopedic Surgery, Rijnstate Hospital, Arnhem, The Netherlands; 2 Department of Orthopedic Surgery, Radboudumc, Nijmegen, The Netherlands; 3 Department of Medical Microbiology and Immunology, Rijnstate Hospital, Arnhem, The Netherlands; 4 Department of Orthopedic Surgery, Sint Maartenskliniek, Nijmegen, The Netherlands

**Keywords:** Total hip arthroplasty, Bacterial contamination, Capsule Sutures

## Abstract

**Background:**

Perioperative preventive measures are important to further reduce the rate of periprosthetic joint infections (PJI) in patients undergoing total hip arthroplasty (THA). During THA surgery, joint capsule sutures are commonly placed to optimize exposure and reinsertion of the capsule. Bacterial contamination of these sutures during the procedure poses a potential risk for postoperative infection. In this exploratory study, we assessed the contamination rate of capsule sutures compared to the contamination of the remains of exchanged control sutures at the time of closure.

**Methods:**

In 100 consecutive patients undergoing primary THA capsule sutures were exchanged by sterile sutures at the time of capsule closure. Both the original sutures and the remainder of the newly placed (control) sutures were retrieved, collected and cultured for ten days. Types of bacterial growth and contamination rates of both sutures were assessed.

**Results:**

Sutures from 98 patients were successfully collected and analyzed. Bacterial growth was observed in 7/98 (7.1%) of the capsule sutures versus 6/98 (6.1%) of the control sutures, with a difference of 1% [CI -6–8]. There was no clear pattern in differences in subtypes of bacteria between groups.

**Conclusions:**

This study showed that around 7% of capsule sutures used in primary THA were contaminated with bacteria and as such exchange by new sutures at the time of capsule closure could be an appealing PJI preventive measure. However, since similar contamination rates were encountered with mainly non-virulent bacteria for both suture groups, the PJI preventive effect of this measure appears to be minimal.

## Introduction

The first preventive strategies to reduce the rate of postoperative infections date from the 1960s and included preoperative antibiotic prophylaxis, laminar air flow in the operating room (OR), and the use of a surgical gown [1, 2]. Subsequent measures focused on reducing the number of colony forming units in the air with laminar flow, effective skin preparation, irrigation of surgical field and sufficient wound closure [3–6]. Despite these measures, periprosthetic joint infection (PJI) after total hip arthroplasty (THA) is still a devastating complication with an incidence of 1–2% [7–12].

Additional perioperative preventive strategies may further reduce the rate of PJI. It has been shown that bacterial contamination is likely to occur during surgery for several materials, such as gloves and suction devices, and repeated exchange is commonly recommended [13–16]. In line with that perspective, hip joint ‘capsule sutures’ are commonly used to expose the femoral neck and to close the capsule after joint replacement [17]. These sutures remain in contact with the patient’s skin during surgery while the skin surface contamination rate in aseptic hip revision surgery can be as high as 13% [18].

We hypothesized that these capsule sutures may be a potential source of avoidable bacterial contamination during THA surgery and that exchange of these sutures at time of closure may further reduce the risk of PJI. In order to explore the potential clinical relevance of this procedure, a short national survey was conducted prior to this study which showed that the capsule sutures are indeed used on a regular basis by the majority (> 60%) of Dutch orthopedic surgeons (unpublished data).

In this study the contamination rate of capsule sutures was established during THA surgery and compared with the contamination of the remains of exchanged control capsular sutures at the time of closure in THA patients.

## Methods

In 100 consecutive patients undergoing primary THA using a posterolateral approach capsule sutures were exchanged with sterile control sutures at the time of closure. Capsule sutures and the remains of control sutures were both cultured for bacterial contamination. All surgeries were performed between April and October 2022 in a high volume (> 1000 arthroplasties/year) teaching hospital by three orthopedic surgeons with more than 10 years of experience in hip surgery.

### Perioperative prevention

In accordance with the hospital’s preoperative *Staphylococcus aureus* eradication protocol, all patients used 4% chlorhexidine gluconate (*Hibiscrub®; SPPH, Quetigny, France)* and intranasal mupirocin 2% nasal ointment 2 days prior to surgery [19]. All patients received a single dose of 2 g of intravenous cefazolin within 60 min before incision. The skin was disinfected using chlorhexidine 0.5% and an iodophor-impregnated plastic incise drape *(Ioban™; 3 M, Saint Paul, MN, USA)* was applied.

### Surgery

**Fig. 1:**
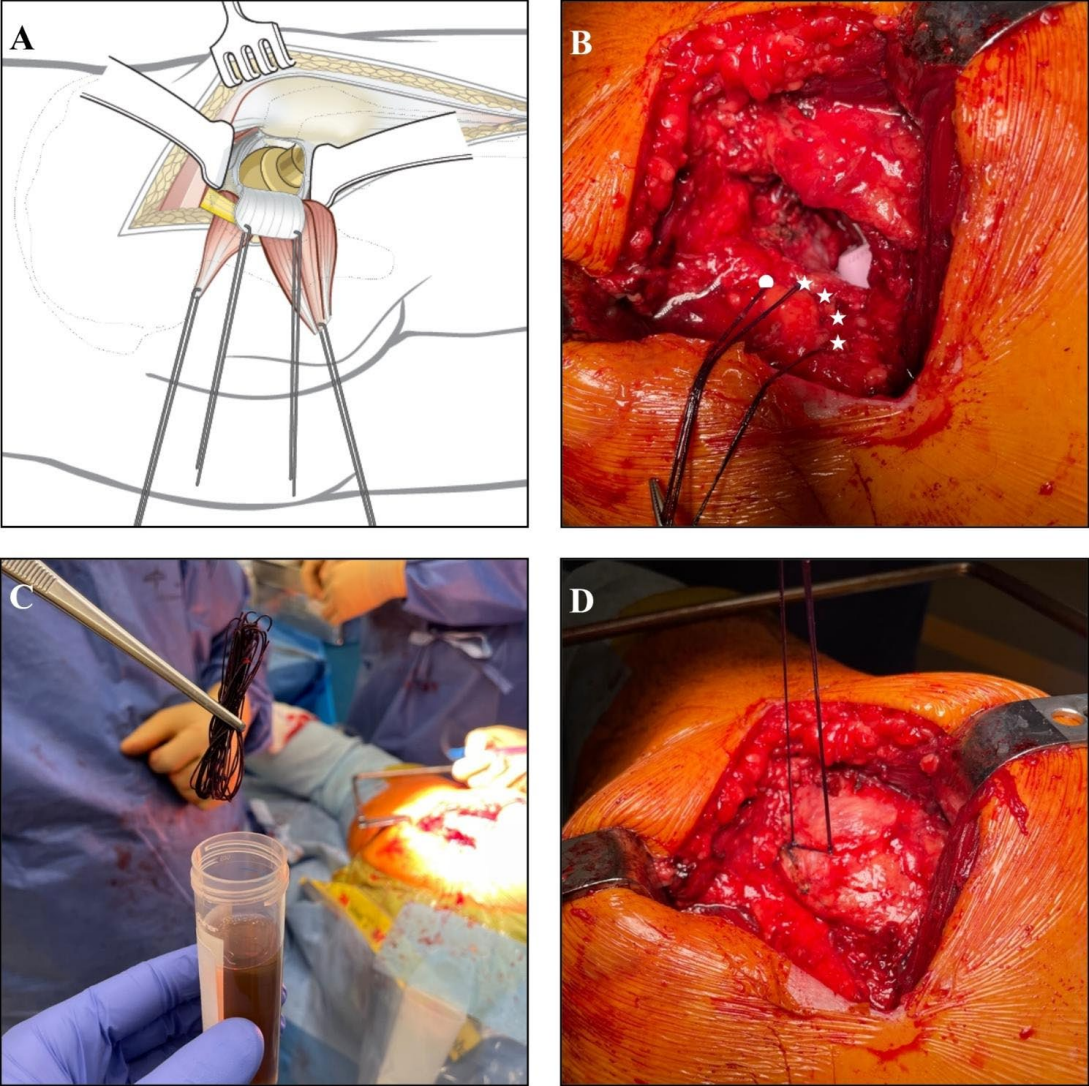
Posterolateral approach of the hip in THA and procedure of suture collection for microbiological culture. **A**: Exposed hip joint by reflecting the short external rotator muscles and the posterior joint capsule using capsule sutures (*image is reproduced with permission from AO Surgery Reference;*www.aosurgery.org); **B**: Hip joint exposed with capsule sutures in situ (• = *piriformis tendon*, ← = *posterior capsule flap*) held by a hemostat. Note that sutures are in contact with the surgical drape; **C**: After removal, original capsule sutures were placed in a sterile container filled with BHI broth; **D**: New, sterile sutures were used for capsule closure, and remains were collected in a separate sterile container

At the time of joint exposure, triclosan (TCS) containing sutures (*Vicryl Plus; Ethicon Inc, Somerville, NJ, USA*) were placed in the hip capsule and the piriformis tendon (Fig. 1A-B). These sutures remained in place during the remainder of the surgery to enhance exposure and protect the sciatic nerve. At the time of closure, after joint replacement, the original sutures were collected using sterile instrumentation and placed in a sterile Falcon 50mL tube filled with 40 mL of Brain Heart Infusion (BHI) broth (*Oxoid, Basingstoke, UK*) (Fig. 1C). Then, gloves where changed and a new set of sterile sutures was subsequently applied to the capsule to replace the original sutures and to reinsert the joint capsule and the piriformis tendon (Fig. 1D). After fixation of the capsule and tendon behind the greater trochanter the excess suture ends were cut and placed in a second sterile tube filled with BHI broth using new sterile instrumentation. These latter sutures served as the control sutures.

### Microbiology

The BHI broths were incubated at 35 ± 1 °C in air for ten days. The BHI broths that became cloudy were inoculated on chocolate agar with Vitox (CHOC; *Oxoid, Basingstoke, UK*) and Fastidious Anaerobe Agar (F.A.A.) with horse blood agar (FHB; *Oxoid, Basingstoke, UK*) and incubated for four days at 35 ± 1 °C in 5% CO_2_ and anaerobic atmosphere, respectively. Subsequently, species determination of cultured bacterial colony forming units (CFUs) on these agars were performed by Matrix-Assisted Laser Desorption/Ionization Time-of-Flight Mass Spectrometry (MALDI-TOF MS; *Bruker, Billerica, MA, USA*). Since an accumulation culture of the used sutures in BHI broth has occurred, no counting of CFUs was performed. If no visible growth was observed after six days, the BHI broths were inoculated by default on CHOC and FHB agars and incubated for an additional four days at 35 ± 1 °C in 5% CO_2_ and anaerobic atmosphere, respectively. Both BHI broths and agar plates were regularly checked for bacterial growth on ten consecutive days. When no bacterial growth on solid agars was detected after ten days, the suture cultures were considered negative.

### Sample size

Previous research has shown that between 10 and 30% of materials used during similar surgeries (e.g., surgical gloves, surgical tools) are contaminated with bacteria [15, 16]. To detect a 15% difference in contaminations between the two types of sutures with a 95% confidence level and 80% power, the recommended sample size was 97 samples per group. The first 10 surgeries served as a pilot study to optimize the suture collection process and subsequent handling at the microbiological laboratory. Sutures collected during these 10 surgeries were analyzed, but their results were not included in the final analysis.

### Data collection and analysis

The following source data were collected from the patients’ electronic medical records: sex, age, body mass index (BMI), preoperative antibiotic use, fixation technique (cemented/cementless), duration of surgery, intraoperative blood loss, and the potential occurrence of a surgical site infection and debridement antibiotics and implant retention (DAIR) in the first 3 months after surgery. The rate of bacterial contamination of capsule sutures was expressed as a percentage and statistical comparison of bacterial contamination rate between capsule and control sutures was performed by calculating a 95% confidence interval (CI). The statistical analysis was performed using SPSS 28.0 (*SPSS Inc. Chicago, IL*).

## Results

Capsule sutures (*n* = 100) and control sutures (*n* = 100) were collected during consecutive surgeries of 100 patients. Sutures collected from two patients were excluded before microbiological culturing due to a protocol violation. The mean age of the patients was 72.4 years (*SD* 8.6) and a total of 69 (70%) were female. In none of the patients’ electronic health records antibiotic use two weeks prior to surgery was reported. In 1 case a DAIR procedure was performed with positive tissue cultures and both negative capsule and control suture cultures. Other patients’ characteristics, including BMI, fixation technique, duration of surgery, and intraoperative blood loss are summarized in Table 1.

**Table 1:** Patients’ characteristics of 98 primary total hip arthroplasties including culture results

	All	Negative suture cultures	Positive capsule and/or control suture cultures*
**N (%)**	98	88	10
**Age**	72.4 ± 8.6	73.1 ± 8.3	67.0 ± 9.8
**Sex**, female, *n (%)*	69 (70)	66 (75)	3 (30)
**BMI**, *kg/m*^*2*^	28.0 ± 4.9	27.6 ± 4.4	30.8 ± 7.4
**Fixation technique**, *n (%)*			
Cemented	67 (68)	60 (68)	7 (70)
Cementless	31 (32)	28 (32)	3 (30)
**Duration of surgery**, *minutes*	68.9 ± 16.9	68.3 ± 17.0	74.3 ± 16.2
Cemented	75.9 ± 13.5	75.6 ± 13.5	79.0 ± 13.5
Cementless	53.8 ± 13.5	52.8 ± 12.8	63.3 ± 19.6
**Intraoperative blood loss**^**†**^, *ml*	339.0 ± 225.3	339.1 ± 231.2	337.5 ± 170.2

All values are means ± standard deviation unless stated otherwise. * Positive cultures in either or both of the capsule or control suture group; ^†^ Intraoperative blood loss was only reported in 48 cases (48%), of which 4 (8%) had positive cultures. *BMI* body mass index.

### Microbiological results

In 10 out of 98 patients the original capsule and/or the control suture cultures were positive for bacterial contamination (Table 2). The rate of bacterial contamination of the original capsule sutures group was 7.1% (7/98) versus 6.1% (6/98) for the control group, with a difference of 1% [CI -6–8]. Overall, 4 out of 7 cases of positive capsule cultures had negative control sutures, whereas 3 out of 6 cases of positive control sutures had negative capsule sutures. In 1 case both the original capsule sutures and the control sutures tested positive for the same micro-organism, whereas in 2 cases positive cultures for both groups with a different micro-organism were found (Table 2). The different micro-organisms identified are summarized in Table 3.

**Table 2:** Analysis of all combinations with positive culture results (*n = 10*)

Culture result	N (%)
1: Capsule suture (+) and control suture (-)	4 (40)
2: Capsule suture (-) and control suture (+)	3 (30)
3: Capsule suture (+) and control suture (+) – *the same micro-organisms*	1 (10)
4: Capsule suture (+) and control suture (+) – *different micro-organisms*	2 (20)

(+): positive culture result; (-) negative culture result

**Table 3:** Isolated micro-organisms found in positive suture cultures in 10 of the 98 patients

No.	Capsule suture	Control suture
**43**	*S. epidermidis* and *Acinetobacter species*	*-*
**51**	*Pantoea species* and *C. acnes*	*-*
**53**	*Pantoea agglomerans* and *C. acnes*	*-*
**79**	* C. acnes* and *Dermacoccus nishinomiyaensis*	*-*
**87**	*Kocuria palustris*	*Micrococcus luteus*
**89**	*Bacillus cereus* complex*	*B. cereus* complex*
**95**	*-*	*Staphylococcus hominis*
**99**	*Staphylococcus warneri*	*Paenibacillus gluconalyticus*
**102**	*-*	*C. acnes*
**104**	*-*	*C. acnes*

‘-‘: no growth; *Morphologically identical *Bacillus cereus* complex was cultured in both groups (both retraction and control suture cultures of this patient showed the same colony morphology of dark grey, dull grey and off-white of *B. cereus* complex).

Three false positive cases were excluded from the analysis because these were classified as laboratory contamination (2 from capsule and 1 from control sutures). In 1 case, one CFU of *Streptococcus mitis* group was cultured at 7 days after inoculation of the BHI broth and 1 day after inoculation of this 6 days incubated broth on solid agars, which is atypical since *S. mitis* group is an easily growing micro-organism in BHI and should have resulted in extensive growth on both CHOC and FHB agars. In 2 cases one CFU of *Cutibacterium acnes* was cultured but only visible at the second and third segment of the FHB agar, respectively, and atypically no visible growth at the first segment with the highest concentration of inoculated BHI.

## Discussion

This exploratory study has assessed the contamination rates of both capsule and control sutures used in 98 patients that underwent primary THA. A negligible difference in frequencies between positive cultures from the capsule (7/98) and control sutures (6/98) was observed.

In this study, the contamination rates of the sutures found are in line with the lower boundaries of the 10–30% contamination rates of other surgical materials such as gloves, surgical instruments and draping material that have been cultured [13–16]. Early literature has reported on experimental studies on the bacterial adherence to different types of surgical sutures [20–24]. More recent literature in arthroscopic surgery also evaluated the contamination rate of sutures. Two studies [25, 26] found a contamination rate of Propionibacterium acnes in arthroscopic rotator cuff repair ranging from 9 to 47% and 12 to 18% respectively, based on different preoperative skin preparations. Another study [27] found a contamination rate of 28.4% in arthroscopic anterior cruciate ligament or meniscal repair. However, none of these studies have included a sterile suture control group. For the interpretation of these high percentages encountered both in earlier studies and in this study the use of control groups is mandatory. To our knowledge, the current study is the first to include a control group when reporting on the contamination rate of surgical material.

Because we found that 7 out of 98 cultured capsule sutures were contaminated, it may be worthwhile to exchange the capsule sutures before capsule closure in addition to the current recommendation to exchange gloves and light handles after a certain period of surgical time [28]. However, this conclusion has become clearly less straight forward from the encountered similar contamination rate of the control sutures which have been exposed to minimal tissue contact only during a very short period of time.

In fact, we feel that it is surprising that the contamination rates of the control sutures were similar to the anticipated relatively high contamination rate of the original sutures. All the samples in the control group were collected using sterile gloves and instruments. In addition, the extent and duration of tissue contact has been limited since sutures were applied before capsule closure and excess of these sutures were retrieved immediately afterwards. Two important questions arise: (1) “How can control sutures obtain similar contamination rates as original capsule sutures with only so limited exposure to surgical environment?” and (2) “Do the positive cultures obtained from the original capsule sutures represent a true bacterial intraoperative contamination?”.

From the results in this study these questions cannot be answered. However, interpretation should be addressed against the fact that to the best of our knowledge no control group was used in the available studies on contamination of surgical materials and instrumentation so far. Still, these studies lead to recommendations in guidelines that promote exchange procedures to reduce the risk of PJI. It is beyond the scope of this study to question the accuracy of the conclusion from these earlier studies, still we feel that the results from our study highlight the need for a control group to improve the validity of conclusions drawn from studies on intraoperative contamination rates of surgical material.

Besides the important strength of a control group in this study, limitations also apply. First, a relatively small sample was used as compared to other infection prevention studies. From the low incidence of PJI it remains difficult to draw conclusions on the actual potential effect of the observed bacterial contamination and the risk of subsequent PJI. In addition, samples were collected from a single center, which may limit the generalizability of the results. On the other hand, since both experienced hip surgeons as well as a microbiology laboratory from a high-volume teaching hospital were involved, we feel our results may be generalizable to comparable hospital settings. Finally, all capsule sutures per group were cultured in a single Falcon tube with BHI broth. Maybe separate culturing of all capsule sutures would have improved differentiation between the possible sources of contamination. For example, with three out of three cultured sutures being positive, it could be argued that the sutures were probably contaminated during the surgery whereas with only one out of three cultured sutures being positive postoperative contamination would be more likely. Future studies need to address this last limitation.

In conclusion, this study showed that around 7% of capsule sutures used in primary THA were contaminated with bacteria and as such exchange by new sutures at the time of capsule closure is an appealing PJI preventive measure. However, since similar contamination rates were encountered with mainly non-virulent bacteria for both suture groups, the PJI preventive effect of this measure appears to be minimal. In particular the observed similar contamination rate in the control group emphasizes the importance of including a control group in intraoperative contamination studies, which has been frequently left behind in earlier studies.

## Data Availability

The data that support the findings of this study are available from the corresponding author, upon reasonable request.

## References

[1] Charnley J (1964). A sterile-air operating theatre enclosure. Br J Surg.

[2] Lidwell OM (1993). Sir John Charnley, Surgeon (1911–1982): the control of infection after total joint replacement. J Hosp Infect.

[3] Parvizi J, Shohat N, Gehrke T (2017). Prevention of periprosthetic joint infection: new guidelines. Bone Joint J.

[4] Alijanipour P, Heller S, Parvizi J (2014). Prevention of periprosthetic joint infection: what are the effective strategies?. J Knee Surg.

[5] Berriós-Torres SI, Umscheid CA, Bratzler DW, Leas B, Stone EC, Kelz RR (2017). Centers for Disease Control and Prevention Guideline for the Prevention of Surgical Site infection, 2017. JAMA Surg.

[6] Swierstra BA, Vervest AMJS, Walenkamp GHIM, Schreurs BW, Spierings PTJ, Heyligers IC (2011). Dutch guideline on total hip prosthesis. Acta Orthop.

[7] Whitehouse JD, Friedman ND, Kirkland KB, Richardson WJ, Sexton DJ (2002). The Impact of Surgical-Site Infections following orthopedic surgery at a Community Hospital and a University Hospital adverse quality of life, excess length of Stay, and Extra cost. Infect Control Hosp Epidemiol.

[8] Sandiford NA, Francescini M, Kendoff D. The burden of prosthetic joint infection (PJI). Ann Jt 2021;6. 10.21037/AOJ-2020-PJI-11.

[9] Premkumar A, Kolin DA, Farley KX, Wilson JM, McLawhorn AS, Cross MB (2021). Projected economic Burden of Periprosthetic Joint infection of the hip and knee in the United States. J Arthroplasty.

[10] Helwig P, Morlock J, Oberst M, Hauschild O, Hübner J, Borde J (2014). Periprosthetic joint infection—effect on quality of life. Int Orthop.

[11] Hegde V, Bracey DN, Johnson RM, Dennis DA, Jennings JM (2021). Increased prevalence of depressive symptoms in patients undergoing revision for Periprosthetic Joint infection. Arthroplast Today.

[12] Abad CL, Haleem A (2018). Prosthetic joint infections: an update. Curr Infect Dis Rep.

[13] Moores TS, Khan SA, Chatterton BD, Harvey G, Lewthwaite SC (2019). A microbiological assessment of sterile surgical helmet systems using particle counts and culture plates: recommendations for safe use whilst scrubbing. J Hosp Infect.

[14] Thaler M, Khosravi I, Lechner R, Ladner B, Coraça-Huber DC, Nogler M (2022). An intraoperative assessment of bacterial contamination on surgical helmets and gloves during arthroplasty surgeries. Hip Int.

[15] Davis N, Curry A, Gambhir AK, Panigrahi H, Walker CRC, Wilkins EGL (1999). Intraoperative bacterial contamination in operations for joint replacement. J Bone Joint Surg Br.

[16] Beldame J, Lagrave B, Lievain L, Lefebvre B, Frebourg N, Dujardin F (2012). Surgical glove bacterial contamination and perforation during total hip arthroplasty implantation: when gloves should be changed. Orthop Traumatol Surg Res.

[17] Petis S, Howard JL, Lanting BL, Vasarhelyi EM (2015). Surgical approach in primary total hip arthroplasty: anatomy, technique and clinical outcomes. Can J Surg.

[18] Dietz MJ, Bostian PA, Ernest EP, Klein AE, LaSala PR, Frye BM (2019). Rate of surface contamination in the operating suite during revision total joint arthroplasty. Arthroplast Today.

[19] Scholten R, Hannink G, Willemsen K, Mascini EM, Somford MP, Schreurs BW (2020). Preoperative Staphylococcus aureus screening and eradication. Bone Joint J.

[20] Katz S, Izhar M, Mirelman D (1981). Bacterial adherence to surgical sutures. A possible factor in suture induced infection. Ann Surg.

[21] Osterberg B, Blomstedt B (1979). Effect of suture materials on bacterial survival in infected wounds. An experimental study. Acta Chir Scand.

[22] Morris MR, Bergum C, Jackson N, Markel DC (2017). Decreased bacterial adherence, Biofilm formation, and tissue reactivity of Barbed Monofilament suture in an. Vivo Contaminated Wound Model J Arthroplasty.

[23] Markel DC, Bergum C, Wu B, Bou-Akl T, Ren W (2019). Does suture type influence Bacterial Retention and Biofilm formation after irrigation in a mouse model?. Clin Orthop Relat Res.

[24] Henry-Stanley MJ, Hess DJ, Barnes AMT, Dunny GM, Wells CL (2010). Bacterial contamination of surgical suture resembles a biofilm. Surg Infect (Larchmt).

[25] Yamakado K (2018). Propionibacterium acnes suture contamination in arthroscopic rotator cuff repair: a prospective Randomized Study. Arthroscopy.

[26] Hong CK, Hsu KL, Kuan FC, Lee YT, Tsai PF, Chen PL (2023). Extended skin cleaning on the shoulder with chlorhexidine reduces the cutaneous bacterial load but fails to decrease suture contamination in patients undergoing arthroscopy rotator cuff repair. J Shoulder Elbow Surg.

[27] Bartek B, Winkler T, Garbe A, Schelberger T, Perka C, Jung T (2022). Bacterial contamination of irrigation fluid and suture material during ACL reconstruction and meniscus surgery: low infection rate despite increasing contamination over surgery time. Knee Surg Sports Traumatol Arthrosc.

[28] Kim K, Zhu M, Munro JT, Young SW (2019). Glove change to reduce the risk of surgical site infection or prosthetic joint infection in arthroplasty surgeries: a systematic review. ANZ J Surg.

